# Facts about the General Medical Care of Adults with Congenital Heart Defects: Experience of a Tertiary Care Center

**DOI:** 10.3390/jcm9061943

**Published:** 2020-06-22

**Authors:** Lavinia Seidel, Kathrin Nebel, Stephan Achenbach, Ulrike Bauer, Peter Ewert, Sebastian Freilinger, Ulrike Gundlach, Harald Kaemmerer, Nicole Nagdyman, Renate Oberhoffer, Lars Pieper, Wibke Reinhard, Linda Sanftenberg, Jörg Schelling, Michael Weyand, Rhoia Neidenbach

**Affiliations:** 1 Department of Congenital Heart Disease and Paediatric Cardiology, German Heart Centre Munich, Technical University Munich, 80636 Munich, Germany; seidell@dhm.mhn.de (L.S.); nebel@dhm.mhn.de (K.N.); ewert@dhm.mhn.de (P.E.); freilinger@dhm.mhn.de (S.F.); kaemmerer@dhm.mhn.de (H.K.); nagdyman@dhm.mhn.de (N.N.); renate.oberhoffer@tum.de (R.O.); 2 Department of Cardiology, University of Erlangen, 91054 Erlangen, Germany; stephan.achenbach@uk-erlangen.de (S.A.); ulrike.gundlach@uk-erlangen.de (U.G.); 3 Competence Network for Congenital Heart Defects, 13353 Berlin, Germany; ubauer@kompetenznetz-ahf.de; 4 Department of Preventive Paediatrics, Department of Sport and Health Sciences, Technical University of Munich, 80992 Munich, Germany; 5 Department of Behavioural Epidemiology, Technische Universität Dresden, 01069 Dresden, Germany; lars.pieper@tu-dresden.de; 6 Cardiology Department, German Heart Centre Munich, Technical University Munich, 80636 Munich, Germany; w.hengstenberg@dhm.mhn.de; 7 Institute of General Practice, University of the Ludwig-Maximilians-University Munich, 80336 Munich, Germany; linda.sanftenberg@med.uni-muenchen.de; 8 Private Group Practice Martinsried, 82152 Martinsried, Germany; prof.dr.schelling@icloud.com; 9 Department of Cardiac Surgery, University of Erlangen, 91054 Erlangen, Germany; michael.weyand@uk-erlangen.de

**Keywords:** adults with congenital heart disease, primary health care, general practitioners, medical health care characteristics in congenital heart defects, real world data

## Abstract

*Background*: Due to the increase in survival rates for congenital heart disease (CHD) in the last decades, over 90% of patients today reach adulthood. Currently, there are more than 300,000 adults with CHD (ACHD) living in Germany. They have an increased need for specialized medical care, since almost all ACHD have chronic heart disease and suffer from specific chronic symptoms, risks, and sequelae. Primary care physicians (PCPs) play a crucial role in referring patients to ACHD specialists or specialized institutions. This cross-sectional study is intended to clarify the real-world care of ACHD from the PCP’s perspective. *Methods:* This analysis, initiated by the German Heart Centre Munich, was based on a 27-item questionnaire on actual ACHD health care practice in Germany from the PCP’s perspective. *Results*: In total, 767 questionnaires were considered valid for inclusion. The majority of the PCPs were general practitioners (95.9%), and 84.1% had cared for ACHD during the past year. A majority (69.2%) of the PCPs had cared for patients with simple CHD, while 50.6% and 33.4% had cared for patients with moderate and severe CHD, respectively, in all age groups. PCPs treated almost all typical residual symptoms and sequelae, and advised patients regarding difficult questions, including exercise capacity, pregnancy, genetics, and insurance matters. However, 33.8% of the PCPs did not even know about the existence of certified ACHD specialists or centers. Only 23.9% involved an ACHD-specialized physician in their treatment. In cases of severe cardiac issues, 70.8% of the PCPs referred patients to ACHD-certified centers. Although 52.5% of the PCPs were not sufficiently informed about existing structures, 64.2% rated the current care situation as either “very good” or “good”. Only 26.3% (*n* = 190) of the responding physicians were aware of patient organizations for ACHD. *Conclusions*: The present study showed that the majority of PCPs are not informed about the ACHD care structures available in Germany. The need for specialized ACHD follow-up care is largely underestimated, with an urgent need for optimization to reduce morbidity and mortality. For the future, solutions must be developed to integrate PCPs more intensively into the ACHD care network.

## 1. Introduction

Congenital heart defect (CHD), the most common isolated congenital organ abnormality, is defined as any type of congenital defect in one or more structures of the heart or blood vessels or a hereditary disorder involving the heart or the great vessels (e.g., Marfan Syndrome or Fabry disease) [1,2]. 

Due to advances in medical care and therapy, the high mortality rate of CHD has been reduced in recent decades, and over 90% of CHD patients now reach adulthood [3,4,5]. It is estimated that over 330,000 adults with CHD (ACHD) live in Germany today, exceeding the number of children with CHD [6]. In time, this trend will strengthen due to a decrease in mortality and a higher age at death of ACHD, especially in patients with complex CHD [7,8].

However, most CHD patients are not completely cured and require medical follow-up or even subsequent “redo-surgery” [3]. All CHD patients, regardless of severity or type, have a chronic heart condition requiring regular follow-up with CHD cardiologists mindful of potential residua, cardiac sequelae, and comorbidities that may seriously affect the patients’ health [9,10,11,12]. The importance of these residual and secondary diseases and the pronounced lifelong need for medical follow-up is illustrated by the significant increase in hospital admissions of ACHD in recent years [13]. In addition, an analysis of the German National Register for Congenital Heart Defects showed that cardiac complications are the main cause of death in ACHD [14]. 

Furthermore, non-cardiac comorbidities play major roles in lifelong ACHD care, but until recently, their importance was clearly underestimated. Neidenbach et al. found that, out of 821 ACHD, over 95% had relevant non-cardiac comorbidities [9]. This finding was supported by Singh et al., a large contemporary study of ACHD hospitalizations (*n* = 255,355) from the US National Inpatient Sample Database [10].

**Figure 1:**
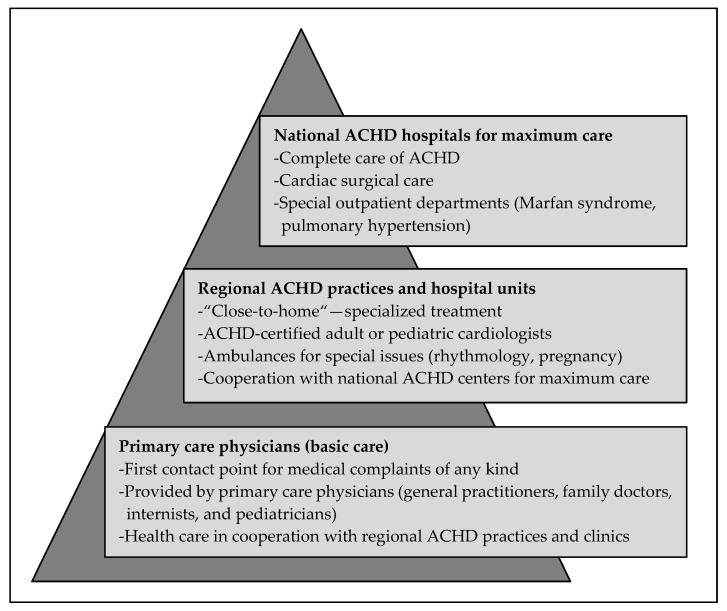
Pyramid of adult congenital heart disease (ACHD) care (modified according to [18]). The basic medical care is provided by primary care physicians (PCPs) who participate in general medical care. They play a crucial role in referring patients to ACHD-specialized institutions. The second level includes regional ACHD hospitals and practices, in which resident adult or pediatric cardiologists care for ACHD and guarantee close-to-home treatment by ACHD-certified cardiologists. At the top, there are national ACHD centers for tertiary care, which also provide cardiac surgical care and special outpatient departments (e.g., for Marfan syndrome, pulmonary hypertension, pregnancy, and genetic counselling).

Whereas a few decades ago, CHD patients’ medical care was predominantly provided by pediatric cardiologists [15], ACHD are now a major adult patient group. In Germany, in the late 1980s, Hannover Medical School and the University Hospital of Cologne were the first centers to provide interdisciplinary care for ACHD on a large scale, following the example of Joseph Perloff in Los Angeles, Gary Webb in Toronto, and Jane Somerville in London [15]. However, the care structures in Germany at that time did not meet the demand sufficiently. Therefore, in 2005, an interdisciplinary working group developed the recommendations and guidelines for optimized ACHD care and physician training that are used today [16]. The ideal care structure was proposed as a pyramid-like system in three stages (Figure 1), with interaction between the three different care levels considered essential [17]. 

Since 2011, 19 supraregional ACHD centers, three regional ACHD clinics, and eight specialized ACHD practices have been accredited by the German Society of Cardiology, the German Society for Paediatric Cardiology, and the German Society for Thoracic and Cardiovascular Surgery. In addition, 349 adult or pediatric cardiologists have acquired an additional qualification for ACHD care [19].

Despite these expansions of care structures, current evidence suggests that ACHD often neglect the need for cardiological follow-up and do not regularly consult ACHD specialists. This leads to a high lost-to-follow-up rate in ACHD, in both Germany and in various other countries [20,21,22]. Indeed, the majority of ACHD sought no follow-up treatment at any German national ACHD center for over 5 years [20]. Similar health care patterns are seen in North America. In the USA, after the age of 19, 42% of patients with CHD refused cardiac follow-up for over 10 years [22]. In Canada, 61% of CHD patients underwent no cardiological follow-up after the age of 18 and 79% of ACHD with complex CHD were in contact only with their PCPs [21].

Recent evidence also suggests that patients mainly want to remain in the care of their PCPs, despite the availability of congenital heart specialists. This can cause major problems as it is essential that the PCP set the right course, referring patients in a timely manner to a CHD specialist for targeted medical care.

The main objective of this study was therefore to evaluate the current care of ACHD in Germany from the perspective of PCPs (detailed definition in Materials and Methods), in order to clarify which counselling needs of ACHD are covered by PCPs and to what extent the existing specialized care structures for ACHD are understood and used by the PCPs.

## 2. Materials and Methods

### 2.1. Setting

The present study was a sub analysis of the nationwide VEmaH registry (www.vemah.info) and is the first large-scale attempt to analyze the real-world health care of ACHD from the perspective of PCPs. The questionnaire-based survey was carried out with a cross-sectional design by the German Heart Centre Munich, Technical University Munich, and the Department of Cardiology, University of Erlangen. These are all large-volume tertiary care centers for ACHD that cover a broad spectrum of almost all types and severity grades of ACHD. The study participants were 2500 PCPs, who had either referred ACHD to the German Heart Centre of Munich in 2018 or were in teaching practices attached to the Technical University of Munich or the Ludwig-Maximilians-University of Munich. 

The definition of a “PCP” in the current survey is based on legal requirements. In Germany, basic medical care is provided by primary care physicians, a generic term for all doctors who participate in general medical care. Originally, the term included the “general practitioner”, a doctor with basic medical training who is usually the first point of contact for the patient in the event of a medical problem. However, this term no longer exists. Instead, the term PCP now includes “specialists in general medicine”, who have completed three years of specialist training; “specialists in internal medicine and general medicine”, who have completed five years of specialist training; and “specialists in internal medicine”, who have completed five years of specialist training and who have decided to work in the field of general medicine. 

### 2.2. Data Collection Procedure and Measurements

Initially, only a low response rate (<15%) was achieved. Through telephone contact, the response rate was eventually increased to 30.7%. Data collection was carried out, with the approval of the Ethics Committee of the Technical University of Munich conferred on 5 April 2017 (157/16 S), using a questionnaire addressed to PCPs (general practitioners, family doctors, and internists) in Germany. Several ACHD experts, PCPs, and epidemiologists, from different federal states in Germany, jointly developed the questionnaire to describe the health care of ACHD from the perspective of the PCPs. The questionnaire comprised 27 questions about the doctor, the practice, and general data about ACHD care. The PCP gave written informed consent before completing the questionnaire. Data were collected and processed in compliance with the relevant federal and state data protection laws. 

### 2.3. Statistical Analysis

Descriptive statistical analyses were performed using IBM SPSS Statistics 23.0 (IBM Inc., Armonk, NY, USA) to characterize the study population. Continuous data were expressed as arithmetic means ± standard deviations (SDs), and nominal and categorical variables were expressed as absolute numbers or percentages. The numbers of valid answers to some questions differed from the total number of study participants. This was due to the presence of multiple answers in some cases and missing data in others.

## 3. Results

Questionnaires filled out by 767 PCPs (34% female) were included in the final analysis. Of these PCPs, 324 stated that their practice was in Bavaria and 29 provided other federal German states as the location for their practice. The remaining 414 (54%) PCPs did not give any information on the federal state in which their practice was located. Of the 767 PCPs, 154 provided information on their age. The mean age of these 154 PCPs was 54.4 ± 8.6 years (range, 23–73 years). The demographic characteristics are presented in Table 1. 

### 3.1. Information Provided by Primary Care Physicians on ACHD Care

According to the PCPs’ reports about their experience with ACHD, 640 (83.4%) of them had cared for ACHD in 2018. However, ACHD accounted for less than one percent of their total patients (Table 2). 

The respondents provided care for ACHD in all age groups. Up to 62.6% cared for ACHD aged 18–64 years, and 27.4%, for patients older than 65 years (Table 2). 

PCPs administered care for almost all types of CHD, even the most complex cases. Furthermore, they treated not only adults with simple CHD but also those with CHD of medium and high severity levels, according to the Warnes classification. 

Nearly all serious complications that typically occur in the long-term course of CHD were encountered by the surveyed PCPs, including threatening cardiac problems such as heart failure, pulmonary hypertension, arrhythmias, and infective endocarditis and also non-cardiac comorbidities, from multi-organ diseases to psychological or intellectual impairment (Table 2). 

### 3.2. Provision of Health Care for ACHD 

**Figure 2:**
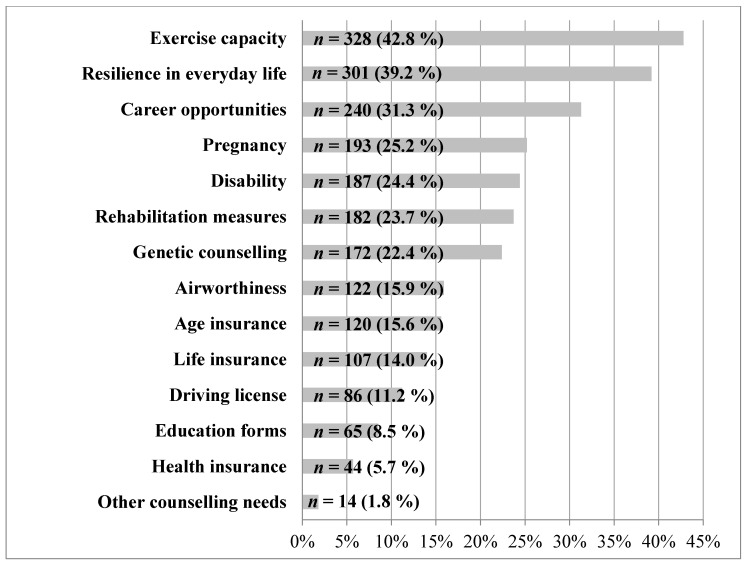
Consultation needs of adults with congenital heart disease, from the primary care physicians’ perspective (multiple answers possible); *n* = absolute number.

With regard to the specific needs of ACHD for advice, the PCP considered, in particular, the physical capacity of the patients, their resilience in everyday life, and medical-, social- and insurance-related issues (Figure 2).

When asked which medical colleagues would be consulted for ACHD treatment, specialized ACHD cardiologists were consulted by only a minority (23.9%, *n* = 183) of PCPs, while the majority (67.0%, *n* = 514) consulted general cardiologists (Table 3). Less than half (48.4%, *n* = 371) of the surveyed PCPs had any knowledge about the existence of certified, ACHD-specialized clinics or centers. Only 20.5% (*n* = 157) of the PCPs were familiar with ACHD-accredited pediatric cardiologists, and only 17.1% (*n* = 131) were familiar with general cardiologists with an additional ACHD certification (Table 3). These replies indicated that only one third of the PCPs (31.3%, *n* = 240) felt adequately informed about the existing ACHD structures (Table 3).

Of a total of 723 responding physicians, only 26.3% (*n* = 190) were aware of patient organizations for ACHD, whereas 67.9% (*n* = 491) were unaware of them. “Don’t know” was chosen by 5.8% (*n* = 42).

## 4. Discussion

Adequate, life-long, specialized health care, provided by experienced ACHD specialists, is one of the most important determinants of well-being and long-term survival in ACHD [6,17,23]. This survey was the first to examine the actual medical care of ACHD in Germany, where nationwide specialized care is available from ACHD-accredited cardiologists and pediatric cardiologists in individual practices, clinics, and centers. 

An estimated 330,000 adults are currently living with CHD in Germany [24]. However, data from 24 accredited ACHD centers indicate that only about 22,000 ACHD are under ACHD-accredited follow-up care [25]. At best, the others are seen by ACHD specialists in private practice or by general cardiologists. This number is also small, however, and over 200,000 ACHD in Germany are thought to lack management by experienced ACHD specialists [4]. This implies a serious deficit, with consequences to be expected, as CHD patients who are not managed by specialists are at risk of inadequate care. This hypothesis is also reflected in the experience of the investigating center.

For the remaining ACHD, not seen in the above-mentioned institutions, primary health care is provided by PCPs as for any other patient. However, PCPs are mostly inexperienced and untrained in dealing with ACHD. Nevertheless, PCPs could and should play decisive roles in referring patients to specialized ACHD facilities. 

### 4.1. Medical Care for ACHD in Germany, from the PCP’s Perspective

This cross-sectional study documents for the first time the care of ACHD currently provided by PCPs in Germany. The response rate for the questionnaire-based survey was quite low; out of 2500 surveyed PCPs, only 767 responded, either primarily or after an additional telephone interview, although nearly 90% cared for ACHD.

This low response rate to the questionnaire, and the comments given in additional telephone interviews, indicates a degree of limited motivation among many PCPs to address the ACHD problem. To some degree, this is not surprising, since ACHD accounts for only a small percentage (less than 1%) of the PCP-managed patient population. However, the number of ACHD in practices may in truth be larger, and physicians may not even be aware that a CHD is present (e.g., the large number of older patients with a bicuspid aortic valve).

According to the questionnaire responses, the PCPs’ patient populations included all types of CHD, from the more common and simpler ones (e.g., septal defects, congenital valve anomalies, aortic coarctation, and tetralogy of Fallot) to rare and sometimes very complex CHD (e.g., transposition of the great arteries, univentricular heart, hypoplastic left heart syndrome, and Eisenmenger syndrome). 

Up to 70% of PCPs currently see CHD patients at medium- or high-severity levels, according to the ACC/AHA-Warnes classification. This situation will likely worsen; an analysis performed by the German National Registry for CHD showed that the prevalence of severe CHD has steadily increased since 2008 [26]. Therefore, ACHD severity in primary care will likely increase in upcoming decades, and many ACHD are likely to suffer from severe CHD residua and sequelae [2].

As indicated in the questionnaire, the numerous and serious typical long-term CHD complications seen by PCPs include life-threatening cardiac problems such as heart failure, pulmonary hypertension, arrhythmia, and infective endocarditis. These all have a major impact on morbidity and mortality in ACHD [14,27,28].

In our experience, this poses a major problem because PCPs usually do not have sufficient knowledge of CHD treatment, residua, sequelae, and associated complications. Given the heterogeneity of ACHD, the diversity of CHD conditions and their different courses, and the large number of possible treatment procedures, it is difficult-to-impossible for untrained physicians to reliably assess the current cardiac status of ACHD and to identify risks at an early stage [29]. This is dangerous, since many medical problems could go unrecognized or not be recognized in time if there is insufficient knowledge of the underlying long-term disease course and possible complications. 

Another issue to bear in mind is that cardiac problems in ACHD often manifest themselves differently from those in acquired heart disease patients. Established treatment regimens for acquired heart defects do not necessarily transfer to CHD [17,28,30].

PCPs’ awareness of these potential and common complications, and routine patient screening for heart disease-specific disorders, is crucial [31]. According to Kaemmerer et al. [18], this is precisely the duty of the PCP in the treatment of CHD. PCPs should be able to assess whether and to what extent current complaints are related to the CHD and, if necessary, refer patients to a more specialized institution, as illustrated in the available pyramid-like ACHD care system (Figure 1). 

If a PCP lacks this awareness, the initiation of diagnostic or therapeutic measures could even endanger the patient. This applies in particular to heart failure, pulmonary hypertension, arrhythmia, aortopathy, and infective endocarditis [10,27,28,30,32,33,34,35,36,37]. 

Unfortunately, from the age range of PCPs alone (mean age 54.4 ± 8.6 years), it can be concluded that they were educated at a time when little was known about ACHD. Even today, this topic is barely represented in medical education, general medical specialization, and continuing education, as the clinical experience of our center and the study leaders confirms. Even most general cardiologists lack sufficient knowledge in the field of CHD. 

Another problem is that given the rapid advance in medical knowledge, it is almost impossible, especially for PCPs, to be up to date on essential information in such a small field. For example, even patients who have had simple shunt lesions (e.g., atrial or ventricular septal defects, or patent ductus arteriosus) successfully repaired at an early stage cannot be considered as completely cured; they may develop relevant sequelae of the underlying disease in later decades [38,39]. This recent observation contradicts current guidelines stating that patients do not require regular follow-up after shunt closure. 

As the questionnaire data demonstrate, non-cardiac problems from multiorgan involvement or psychological or intellectual impairment, which increase with age in ACHD, are another major concern. These expectations are consistent with the findings of Baumgartner (2014), who reported that ACHD over age 60 are a small but increasing proportion of CHD patients [6]. In the next few years, the number of ACHD over age 65 will increase significantly as treatment approaches advance with improvements in congenital cardiac surgery [23,40].

This is in line with recent studies showing that almost all ACHD are affected by non-cardiac comorbidities that influence the long-term course of the CHD, in particular, metabolic disorders (hyperlipidemia and hyperuricemia), thyroid dysfunction, and hepatic, nephrologic, and neurological diseases [9,10]. For women, gynecological and obstetrical questions become especially relevant [12,13,41]. Moreover, psychological and intellectual limitations are also frequent [42]. It is of great importance that PCPs recognize these non-cardiac comorbidities and consider, on the one hand, how CHD influences the comorbidities (e.g., anemia, iron deficiency, and hyperuricemia from cyanotic heart defects) but, on the other hand, how the comorbidities can affect CHD (e.g., additional coronary artery disease).

Regarding the specific need for advice for ACHD, PCPs considered, in particular, patients’ physical capacity, resilience in everyday life, and medical-, social-, and insurance-related issues (Figure 2). To answer these questions without being deeply involved with ACHD as a specialty seems almost impossible. Since PCPs in many cases lack sufficient clinical experience with CHD, familiarity with the available support structures for ACHD care in Germany, including to whom they can refer such patients, is very important. Unfortunately, this familiarity is not the norm; fewer than half (48.4%, *n* = 371) of the surveyed PCPs had any knowledge about the existence of certificated, ACHD-specialized clinics or centers. Moreover, ACHD-accredited pediatric cardiologists were known to only 20.5% (*n* = 157), and general cardiologists with an additional ACHD certification, to only 17.1% (*n* = 131). 

The lack of information on ACHD care extends to knowledge about patient organizations; 67.9% of PCPs stated that they did not know of any such groups. This is regrettable, as it is helpful for patients to discuss lifestyle issues with other affected people. Prominent among these are social or occupational issues and pension or disability issues.

### 4.2. The Consequences of Deficiencies in ACHD Care

Because all patients with CHD are chronically ill, all affected patients have a special need for lifelong surveillance and counselling so that problems can be recognized early on and corrected [17]. Lapses in care lead to increased morbidity and mortality in ACHD, so it is important that ACHD regularly participate in specialist follow-up or prevention programs [43]. Monitoring intervals depend on the type and severity of the heart defect and can vary from weeks to several years. In addition, PCPs are uniquely positioned to encourage ACHD to adopt better health behavior. This includes highlighting the importance of exercise, healthy nutrition, and mental health for disease prevention, starting with younger ACHD. However, as according to our data, CHD centers and ACHD specialists are often not even known by PCPs to exist, this unfortunately does not always take place. This may result in an alarming loss-to-follow-up, a worldwide problem, as studies from various countries and continents confirm [4,20,21,22,44].

The reasons for loss-to-specialist-follow-up are diverse, including factors related to patients, health care providers, economics, inadequate patient and family preparation for transition, cognitive and/or psychosocial impairments, patient–provider attachment, and inadequate program integration [43]. Other reasons are that the patients feel well, that they want to be free, or that they are not even aware of their CHD (if not reported by the parents). Loss-to-specialist-follow-up typically occurs when patients have to leave a pediatric cardiology setting and enter an internal-medicine or adult-oriented one [44]. It is in precisely this area that PCPs could intervene and provide ACHD with adequate follow-up care if the PCP understood the problem and knew where best to refer the patient. The awareness of PCPs and patients must therefore be further enhanced.

For this purpose, it is necessary to draw the attention of other medical disciplines to the particularities of ACHD. This applies especially to the fields of internal medicine (including pulmonology, hematology, nephrology, and hepatology), obstetrics, human genetics, neurology, dentistry, and occupational/sports/social medicine, as well as psychology and psychosomatics.

As a direct result of our present study, awareness campaigns have already been started throughout Germany. They are being carried out with the support of German cardiac societies, patient organizations (e.g., Deutsche Herzstiftung, Herzkind e.V), and, in some cases, even with the support of the investigative pharmaceutical industry (e.g., Janssen-Actelion). Moreover, other professional groups are increasingly becoming interested in the topic. These include physicians specialized in general medicine, internal medicine, gynecology and obstetrics, genetics, psychology and psychiatry, sociology, and nutritional and sports medicine. The number of colleagues turning to us for advice has risen considerably.

In practice, awareness campaigns occur within the framework of scientific seminars at conferences and congresses and at regional or national information events for doctors and patients. In addition, the public media (television, the press, and the Internet) have been and are presently involved in the campaigns.

### 4.3. Study Limitations

The study is limited by the selection of the study population, since many of the included PCPs were physicians who had referred patients to the German Heart Center Munich (supra-regional ACHD center) or physicians who were part of the university network of the Technical University Munich and the Ludwig-Maximilians-University-Munich. It can be assumed that these referring physicians are quite familiar with ACHD patients and their existing care structures, as they frequently referred their patients to an ACHD center themselves.

In addition, a selection bias should be considered, as it is not possible to investigate why some physicians agreed to fill out the study questionnaire while others rejected it. It can be assumed that physicians who are interested in the ACHD topic were more likely to participate in the study. By contrast, physicians who were not familiar with ACHD may have completed the questionnaire less frequently. Therefore, it is possible that, in reality, PCPs are even less aware of the existing ACHD care structures than the present study data indicate.

Furthermore, our study showed geographical limitations, as the majority of the participating PCPs were resident in Bavaria. The generalization of the conclusions to the primary care of ACHD throughout Germany or other countries is therefore only possible to a limited extent. In Germany, an extension of the study to other federal states has been initiated on the basis of the data collected in the present study. Other ACHD centers, located at the university hospitals of Cologne, Hamburg, Mannheim, or Tübingen, are therefore now actively participating and others are committed to follow.

## 5. Conclusions

The results of this work show that there are still considerable deficits in medical care for ACHD in Germany. Although a nationwide medical care network of certified general cardiologists, pediatric cardiologists, and specialist clinics is available, the majority of PCPs are insufficiently informed about existing ACHD care structures. Although there are regional and nationwide ACHD specialists and centers, they are not yet sufficiently known and used.

The willingness of PCPs to dedicate their attention to the ACHD problem is currently unsatisfactory. Moreover, the need for lifelong specialized ACHD follow-up and care is underestimated, despite a higher-than-expected long-term complication rate, even for simple and corrected CHD. Therefore, to reduce morbidity and mortality, it is essential to further raise awareness of the needs of ACHD and to optimize medical health care accordingly.

This study is intended to contribute to the development of further concepts for increasing the visibility of ACHD care needs among PCPs. Awareness campaigns for patients and physicians are one step. Another is the improvement of the cooperation between the various levels of Germany’s extant ACHD health care pyramid; this would serve to improve the care of ACHD through interdisciplinary management.

It is to be expected that this problem will be found to be even worse in other countries where there is a less advanced infrastructure for caring for ACHD.

## Figures and Tables

**Table 1:** Socio-demographic variables of the surveyed primary care physicians (*n* = 758, PCPs with missing surveys = 9).

	**Mean ± SD**	**Range**
Age in years	54.4 ± 8.6	(23–73)
Years of employment as a PCP	18.0 ± 10	(0–45)
**Sex**	***n***	**%**
Male	503	66.4
Female	255	33.6
**Specialization (multiple answers possible)**	***n***	**%**
General practitioner (PCP)	733	95.6
Other specialization	56	7.3

Abbreviations: PCP = primary care physicians, including “general practitioner”, “specialists in internal medicine and general medicine”, and “specialists in internal medicine”, who had completed specialist training in the field of general medicine; *n* = number; SD = standard deviation.

**Table 2:** Primary care physicians’ (PCP) reports (*n* = 767) on their ACHD patients.

**PCPs cared for ACHD?**	***n* (%)**
Yes	640 (83.4)
No	114 (14.9)
No awareness	7 (0.9)
Missing data	6 (0.8)
**Proportion of ACHD in PCP practices, in relation to their entire patient collective**	***n* (%)**
<1%	575 (75.0)
1–10%	64 (8.3)
>10%	1 (0.1)
No awareness	9 (1.2)
Missing data	118 (15.4)
**Number of PCPs who cared for ACHD, by age distribution (multiple answers possible)**	***n* (%)**
18–34 years	445 (58.0)
35–64 years	486 (63.4)
> 65 years	210 (27.4)
**Number of PCPs who cared for ACHD, by CHD severity (according to [11], multiple answers possible)**	***n* (%)**
Simple	388 (50.6)
Moderate	531 (69.2)
Severe	256 (33.4)
**Number of PCPs who cared for ACHD, classified into the types of congenital heart defects (multiple answers possible)**	***n* (%)**
Atrial septal defect	327 (42.6)
Aortic valve stenosis/insufficiency	281 (36.6)
Ventricular septal defect	249 (32.5)
Coarctation of the aorta	220 (28.7)
Tetralogy of Fallot	192 (25.0)
Transposition of the great arteries	165 (21.5)
Atrioventricular septal defect	152 (19.8)
Pulmonary valve stenosis/insufficiency	137 (17.9)
Persistent ductus arteriosus Botalli	130 (16.9)
Other congenital heart defect	89 (11.6)
Hypoplastic left heart syndrome	27 (3.5)
Univentricular heart	24 (3.1)
**Number of PCPs who cared for ACHD, with specified comorbidities (multiple answers possible)**	***n* (%)**
Cardiac arrhythmia	361 (47.1)
Heart failure	332 (43.3)
Psychological or intellectual impairment	234 (30.5)
Pulmonary (arterial) hypertension	154 (20.1)
Neurological complications	92 (12.0)
Thromboembolism	72 (9.4)
Coronary artery disease	72 (9.4)
Haematological disorders	69 (9.0)
Others	41 (5.3)
Infective endocarditis	34 (4.4)
Coagulation disorders	28 (3.7)
Sudden cardiac death	22 (2.9)

Abbreviations: n = number; ACHD = adults with congenital heart disease, CHD = congenital heart defect.

**Table 3:** Characteristics of the health care of adults with congenital heart disease. Multiple answers possible.

**Number of indicated medical colleagues involved in the treatment of ACHD (multiple answers possible)**	***n* (%)**
General cardiologist	514 (67.0)
Pediatric cardiologist	201 (26.2)
ACHD-specialized physician	183 (23.9)
others	152 (19.8)
**Knowledge of PCPs about existing specific ACHD care structures**	
Certified ACHD-specialized clinics/ACHD centers	371 (48.4)
Established pediatric cardiologists with an ACHD certification	157 (20.5)
General cardiologists with an ACHD certification	131 (17.1)
Missing data	108 (14.0)
**PCPs’ answers on whether they feel sufficiently informed about existing ACHD structures**	
Not sufficiently informed	372 (48.5)
Sufficiently informed	240 (31.4)
Do not know	110 (14.3)
Missing data	45 (5.8)

Abbreviations: *n* = absolute number; ACHD: adults with congenital heart disease; PCPs: primary care providers.
